# Numerical modelling and experimental validation of an orifice plate-based hydrodynamic cavitation process for improving the biomass pretreatment

**DOI:** 10.1016/j.btre.2025.e00925

**Published:** 2025-09-12

**Authors:** Shetal Roy, Souman Rudra, DI Muhandiram, MM Roshid

**Affiliations:** a Department of Engineering Sciences, University of Agder, Grimstad 4879, Norway; b Department of Mechanical Engineering, CUET, Bangladesh

**Keywords:** Hydrodynamic cavitation reactor, Multiple hole orifice plate, Cavitation number, Transient multiphase model

## Abstract

Hydrodynamic cavitation can be used to pretreat biomass by utilizing the energy released during the collapse of cavitation bubbles. Delignification as pretreatment enhances the biodegradation of lignocellulosic composition. The present study employed a CFD model and experimental validation of the numerical analysis of hydrodynamic cavitation (HC) in multi-hole orifice (MHO) designs under various operating conditions. The total 14 geometry of the orifice plate depends on the plate thickness, the number of holes, and the hole orientation used to analyze and optimize geometry for the biomass pretreatment process. Three phases are used in numerical analysis and experimentation to investigate the effect of particles on cavitation. The simulated results regarding velocity and pressure gradients, turbulence quantities, and vapor volume fractions are critically analyzed and discussed. The cavitation number changes to 0.22 in the experiment and 0.16 in the simulation due to the presence of biomass particles. Orifice plate thickness was found to significantly influence cavitation inception and evolution. 4 mm thickness and nine holes with specific orientations were found to be an optimized geometry with the lowest cavitation number, maximum pressure drop, and highest throat velocity. In the experimentation, 0.8 mm biomass particles were used in the mixture (2 % w/w) to determine the effect of biomass particles on the flow. This result helps identify the critical operating and design parameters and the impact on the cavitation of particles to achieve the desired cavitation phenomena.

## Highlights

• CFD and experiments studied hydrodynamic cavitation in multi-hole orifice(MHO) designs.
• Optimized MHO design: 4 mm thick, 9 holes, case 5 showed best cavitation(Cv).
• Case 5 had min Cv (0.44), max turbulence, and highest vapor fraction in CFD.
• Biomass (0.8 mm, 2 % w/w) increased Cv from 0.46 to 0.59, reducing cavitation.
• Experimental Cv for biomass mixture was 0.93, confirming lower cavitation.

## Graphical abstract

**Figure:**
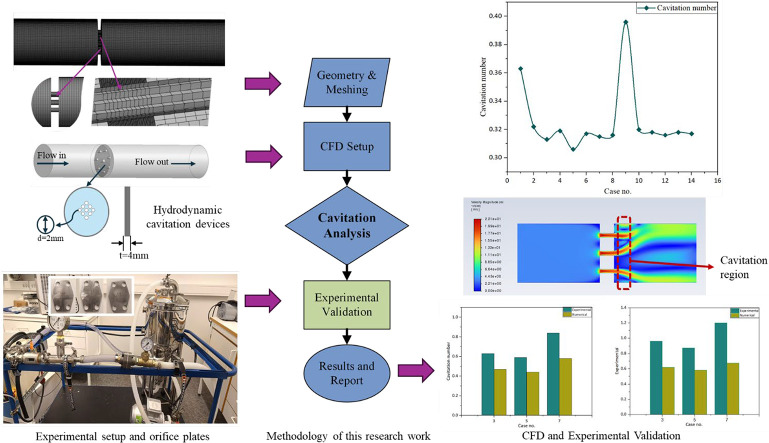

## 1 Introduction

The whole world faces a severe challenge in achieving energy savings targets, and in the search for new renewable energy sources, extensive research has been conducted to develop biotechnological techniques that enable the use of biomass residue with lignocellulosic composition to manufacture new goods, including biofuels, biomaterials, and chemical products [1,2]. The hydrodynamic cavitation (HC) process is one of the most promising industrial-scale applications due to its high synergy effect with other physical and chemical processes, robust scalability, and low cost.

Hydrodynamic cavitation reactors improve processes such as biomass pretreatment and wastewater treatment by utilizing hydroxyl radicals, localized hot spots, and severe shear for various physicochemical transformations [3]. The reactor's operational principle is as follows: as the liquid flows through a constriction, the liquid velocity rises while the static pressure drops, corresponding to the energy equation. At this point, the liquid flow collapses into several areas of weak regions, which manifest as pollutants and gas bubbles. These free radicals will further trigger micro-combustion reactions, chemically damage the cellulose walls of biomass [4,5]. The goal of all types of cavitation is to heat liquids, especially water, or to undergo physical and chemical changes like homogenization, emulsion, gas dissolution, degassing, and decanting of solid particles; to improve catalysts and speed up chemical reactions like esterification and transesterification, especially in the processes used to produce biodiesel [6].

Employing hydrodynamic cavitation has one of the main advantages of reducing the use of hazardous solvents. This aligns with the growing need for environmentally friendly processing methods as businesses seek to reduce their environmental impact. Energy efficiency, a crucial aspect of industrial food preparation, is a key characteristic of HC. Traditional sterilization techniques are energy-intensive, resulting in higher operating expenses [7,8]. Several studies about cavitation generation are listed in Table 1.

**Table 1:** Related research work on the hydrodynamic cavitation reactor.

CR type	Analysis type	Methodology	Studied parameters	Objective function	Ref.
Orifice plate & Venturi tube	CFD	3-D flow, Mixture model & k -ε Turbulence model	Inlet pressure, Density, Vapor fraction	Geometry optimization	[5]
Orifice plate	CFD & Experimental	3-D flow, Mixture model & SST k -ε Turbulence model	Pressure, Throat velocity	Ethanol production	[9]
Orifice plate	CFD	2-D flow & K-ω Turbulence model	Velocity and pressure gradients, VVF, and turbulence quantities	Geometry optimization	[10]
Orifice plate	CFD	3-D flow, Mixture model & SST k-ω Turbulence model	Turbulence, pressure ratio, velocity, vapor fraction	Geometry optimization for water treatment	[11]
Orifice plate	CFD	2-D flow, VOF method & SST k-ω Turbulence model	Pressure, Velocity, vapor fraction	Geometry optimization	[12]
Orifice plate	Experimental	N/A	Cavitation number, Degradation, Flow rate	Wastewater treatment	[13]
Venturi tube	CFD	2-D flow, Mixture method, SST k-ω Turbulence model	Pressure, Velocity, Vapor fraction, Time step	Wastewater treatment	[14]
Venturi tube	CFD and Experimental	3-D flow, Mixture, k-ε Turbulence model	Pressure, Velocity, Vapor fraction	Biomass pretreatment	[15]
Venturi & Orifice plate	CFD	3-D flow, Mixture method, k-ε Turbulence model	Pressure, Density, Velocity, Vapor fraction	Geometry optimization	[5]
MHO	CFD	3-D flow & Mixture method	d/D ratio, Turbulence intensity, Vapor fraction,	Geometry optimization	[4]
Nozzle	CFD and Experimental	2-D flow, Mixture method, k-ω Turbulence model	Turbulence intensity, Pressure, Velocity, Vapor fraction	Effects of Solid Particles on Cavitation	[16]

*OP= Orifice Plate, VT=Venturi Tube, CR= cavitation Reactor, Angle= Divergence angle of the Venturi tube, SHO= Single Hole Orifice, and MHO= Multiple Hole Orifice.

In the hydrodynamic cavitation (HC) system, energy is released when cavitation bubbles collapse and implode. This intense energy causes the dissociation of water molecules in the solution, generating reactive hydroxyl radicals (•OH) and hydrogen radicals (•H). The highly reducing •H radicals readily react with dissolved oxygen (O₂) to form (•O₂⁻), which, along with •OH radicals, are powerful oxidizing agents. These reactive species play a critical role in the degradation of organic contaminants in the solution. The oxidation reactions are also exothermic, contributing to a significant temperature increase in the solution, which may further enhance the reaction kinetics and overall treatment efficiency [17]. The following three reactions are part of the HC effect's action mode in the breakdown process of organic pollutants:

H_2_O + Hot spots (from HC) **→** •*H*^+^ + •OH^-^

•OH^-^**→** H_2_O_2_**→** H_2_O + O_2_

O_2_ + •*H*^+^**→** •O_2_ + *H*^+^

**Fig. 1:**
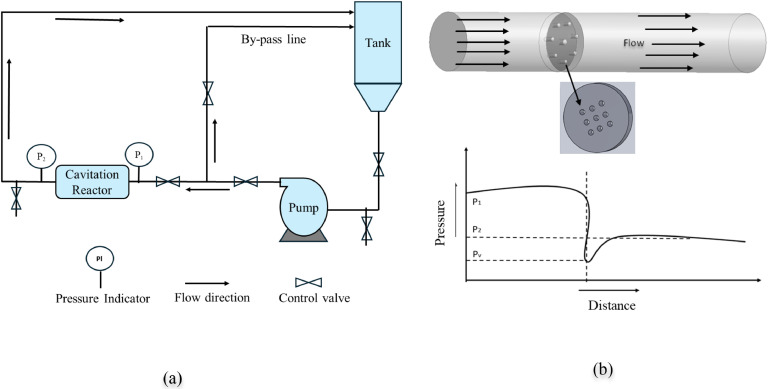
(a) Hydrodynamic cavitation generation unit. (b) Orifice plate as a Cavitation reactor and Pressure drop.

Fig. 1(a) shows the hydrodynamic cavitation (HC) generation unit comprising several integrated components. Despite its numerous advantages over conventional biological oxidation processes, the widespread daily use of industrial-scale HC remains limited, primarily due to its comparatively higher operational costs [15].

One of the most used tools for hydrodynamic cavitation is the orifice. Orifice plates may have single or multiple holes. An increase in kinetic energy, pressure drop, and a proper cavitation yield are all provided by the right arrangement and number of orifices [6]. Figure 1(b) illustrates the orifice plate used as a cavitation reactor and the associated pressure drop across the flow. An increase in the number of holes enhances turbulence intensity, significantly influencing the cavitation process—particularly in applications such as delignification and water treatment, where improved cavitation intensity translates to higher process efficiency. Additionally, the plate's thickness and the holes' orientation play a crucial role in modulating cavitation behavior within the reactor.

Hydrodynamic cavitation is a potential technology for various applications, including dye degradation, sludge treatment, biodiesel production, disinfection, and the breakdown of emerging organic contaminants for use in pharmaceuticals, wastewater treatment, and to enhance drug solutions and biodegradability [18,19]. It effectively disrupts the lignin–carbohydrate matrix within lignocellulosic biomass, thereby enhancing the efficiency of subsequent processes such as enzymatic hydrolysis. Owing to its well-documented energy efficiency and environmentally benign nature, HC presents a promising approach for large-scale biomass processing. Delignification, a predominantly oxidative process, involves the removal of lignin from plant materials through a range of biological and physicochemical methods [20]. Conventionally, lignin is extracted from lignocellulosic biomass using physical and chemical pretreatment methods involving acidic, alkaline, or green liquor-based chemicals. The structural rigidity of lignin is primarily attributed to intramolecular hydrogen bonding formed by its hydroxyl groups, which reinforces its resistance to degradation [21].

Extremely reactive radicals, OH^-^ and superoxide (O_2_), are produced during the reaction in the HC process. This intermediary molecule breaks down lignin through ring opening, side chain removal, and demethylation [22]. The interaction between HOO- and H_2_O_2_, which is created by the recombination reaction of OH^-^ in alkaline circumstances, produces the radicals OH^-^ and O_2_. The kinetic energy per unit mass caused by eddies in turbulent flow is known as turbulence kinetic energy (TKE). Convective and mechanical sources of turbulence add up to TKE [23]. A key concept in turbulence study is turbulent kinetic energy, which is the energy involved in chaotic fluid motion and how it dissipates in non-breaking waves. It shows that shear in wave orbital velocities causes fluid turbulence .

Recent studies on cavitation have extensively focused on cavitation phenomena numerically or experimentally only for water. These approaches have not considered the third phase in the case of biomass pretreatment, which is essential to know how the presence of biomass changes the cavitation number in numerical analysis and experimentation. This study investigated the change in cavitation numbers due to biomass in water and the application of hydrodynamic cavitation reactors (HCRs) as an environmentally friendly and cost-effective technology for biomass pretreatment and delignification. This was achieved by designing, simulating, and experimentally validating a numerical model of an HCR, focusing on the influence of geometric parameters and cavitation behavior. The comparison of both water and biomass-water mixture in numerical and experimental validation could be helpful for further studies on the efficient separation of lignin using HCR.

## 2 Methodology

### 2.1 Numerical simulation

The required quantities are found using ANSYS Fluent as 2024 R1™ (Research-Ansys Academic Multiphysics Campus Solution), a commercial CFD program. Using an established turbulence model (k-model) with the standard viscous flow equations controlling the transport of phases (Eulerian multiphase) or mixtures (Mixture model) forms a basic two-phase cavitation model. The transient flow iteration was done with a time step of 0.00001 s to make the vapor phase visible in the cavitation model. This section describes the mathematical governing equations of the cavitation phenomena.

#### 2.1.1 Conservation equation

The Eulerian model solves the continuity equation for every phase individually, with the number of solving equations equal to the number of phases. The continuity equation [24] for the phase is written as:

(1) $$\left(\frac{\partial }{\partial t}\rho_{m}\right)+\nabla \cdot \left(\rho_{m}{\vec{u}}_{m}\right)=0$$

Where $\rho_{m}$ is the mixture density, & ${\vec{u}}_{m}$ is the mass-averaged mixture velocity. The momentum equation for the mixture flow is written as [25]:

(2) $$\left(\frac{\partial }{\partial t}\rho_{m}{\vec{u}}_{m}\right)+\nabla \cdot \left(\rho_{m}\vec{u}{}_{m}\vec{u}\cdot m\right)=\nabla p+\nabla \cdot \left[\mu_{m}\left(\nabla {\vec{u}}_{m}+\nabla {\vec{u}}_{m}T\right)\right]+\rho_{m}\vec{g}+\vec{F}$$

Where ${\vec{u}}_{m}$ is the velocity vector, $\mu_{m}$ is the viscosity, $\rho_{m}g$ is the gravitational body force, and the term *F* accounts for additional external body forces applied to the fluid volume [10]. $\vec{F}$ may arise from the interaction with dispersed phases.

#### 2.1.2 Turbulence model

Although several turbulent models are available, the Realizable k-ε model was initially employed to simulate this flow situation. This model estimates the turbulent kinetic energy and viscous dissipation rates to determine the turbulent viscosity in the flow field [26]. k-ε turbulence model is described below, k-ε turbulence model:

(3) $$\frac{\partial \left(\rho k\right)}{\partial t}\;+\Delta \;\left(\rho kU\right)=\Delta \left(\frac{\mu_{t}}{\sigma_{\varepsilon }}\;\Delta .k\right)\;+\;2\mu_{t}E_{ij}E_{ij}--\rho \varepsilon$$

(4) $$\frac{\partial \left(\rho \varepsilon \right)}{\partial t}\;+\;\Delta \left(\rho \varepsilon U\right)\;=\;\Delta \left(\frac{\mu_{t}}{\sigma_{\varepsilon }}\Delta .\varepsilon \right)\;+C_{1\varepsilon }\frac{\varepsilon }{k}\;2\;\mu_{t}E_{ij}E_{ij}-C_{2s}\rho \frac{\varepsilon^{2}}{k}$$

$$\mu_{t}=C_{\mu }\rho \frac{k^{2}}{\varepsilon }\;E_{ij}=\;\frac{1}{2}\;\left(\frac{\partial U_{i}}{\partial X_{j}}\;+\;\frac{\partial U_{j}}{\partial X_{i}}\right)$$

Where, *k* =turbulent kinetic energy, ε = dissipation rate, $\mu_{t}$ = eddy viscosity and Constants are-

$$C_{\mu }=0.09,\;C_{1\varepsilon }=\;1.44,\;\sigma_{k}=1,\;C_{2\varepsilon }=\;1.92,\;and\;\sigma_{\varepsilon }=1.3.$$

The governing equation of the vapor transport in terms of vapor fraction (*f*) is given by [5]:

(5) $$\frac{\partial }{\partial_{t}}\left(\rho m\;f\right)+\Delta .\left(\rho mnn\;f\right)=\Delta .\left(g\Delta f\right)+Re+Rc$$

Where *ρ*_m_ is the mixture density, *f* is the vapor fraction, *g* is the coefficient of the effective exchange, and V_v_ is the velocity of the vapor phase. *R_e_* and R_c_ are the vapor generation and condensation rates (or phase change rates), respectively [5]. The square root of turbulent kinetic energy (√k) has the same magnitude of the relative velocity.

(6) $$P<\;P_{sat},\;R_{e}=C_{e}\frac{V_{ch}}{\sigma }\;\rho l\;\rho v\sqrt{\frac{2\left(Pv-P\right)}{3\rho_{l}}}\left(1-f\right)$$

(7) $$P>P_{sat},R_{e}=C_{c}\frac{V_{ch}}{\sigma }\;\rho l\rho l\;\sqrt{\frac{2\left(p-Pv\right)}{3\rho_{l}}}\;f$$

Where, V_ch_ = Characteristic turbulent velocity, σ = Coefficient of the surface tension, P_v_ = Vapor pressure, P_sat_ = Saturated vapor pressure, and two constants are C_e_ = 0.02 and C_c_= 0.01.

The turbulent pressure fluctuation is estimated by calculating the threshold pressure change, which is defined by

$$P_{v}\;=\;\left(P_{sat}+\;\frac{P_{tur}}{2}\right)$$

Here, *P_tur_*= 0.39*pk*

#### 2.1.3 Boundary conditions

In all cases, the flow has been initialized from the inlet. The pressure of the liquid under consideration is specified at the inlet of the pipe. A stationary, no-slip boundary and Surface tension of water 0.072 N/M are imposed on the pipe's wall. The governing equation's solution can be obtained for the given geometrical configuration and boundary conditions. When choosing a border condition, the cavitation number, which depends on throat velocity, vapor pressure, and downstream pressure, is significant for given boundaries. Since there was no cavity at the entrance, the Vapor Volume Fraction (VVF) was initially assumed to be zero, and the flow is considered transient flow in this study. The flow through the orifice plate is transient because the upstream or downstream parameters change with time. Table 2 presents the numerical assumptions taken in the Ansys Fluent simulation.

**Table 2:** Numerical schemes.

Model	Numerical scheme	Cavitation model	Zwart model
Solver	Pressure-Based	Viscous model	Realizable k-ε
Pressure-velocity coupling	Coupled	Turbulence Multiphase Model	Mixture
Velocity	Absolute	Temperature	300K
Gravity	−9.81 ms^-1^ (y direction)	Operating pressure	101,325 Pa
Model	Eulerian	Volume Fraction	First order upwind
Number of phases	Three	Momentum equation	First order upwind
Primary phase	Water- liquid	Flow	Transient
Secondary phase	Water-vapour	Gradient	Least Squares Cell Based
Granular phase	Wood particles	Energy	First order upwind

#### 2.1.4 Meshing of the model

The present CFD study employed a structured mesh (Figure S1) and its associated metrics, including a maximum skewness of 0.72 and high smoothness with a transition ratio of 1.2. Finer cells in the flow passage and denser cells close to the walls were used in the grid design to capture the boundary layer and accurately simulate the generation of vapour/bubbles. In this study, a structured mesh with defeaturing has been employed to improve the accuracy of the results.

The fluid volume has been used with a multiphase model. Water-liquid, water-vapor, and wood (Oak) particle phases have all been employed. Fig. 4 displays the equipment geometry and the computing grid. Table 4 presents the geometrical details of different Orifice plates used in this numerical analysis. To ensure the pressure is recovered as per ISO 5167–2:2003 in the downstream flow [11], two pipes of 4 × *D* and 6 × *D* lengths were added upstream and downstream of the MHO plate. The properties of the phases are tabulated below Table 3.

**Table 3:** Properties of the phases in the multiphase model.

Parameter/Phase	Water-liquid	Water-vapor	Wood particle	Unit
Density	998.2	0.5532	400	kg/m3
Thermal conductivity	0.6	0.026	0.054	W/m.K
Specific heat	4182	Polynomial	1500	J/kg.K

#### 2.1.5 Cavitation number

Hydrodynamic cavitation (HC) 's efficiency in pretreatment of lignocellulosic biomass strongly depends on identifying and understanding key operational parameters that govern the underlying physical and chemical mechanisms. Among these, cavitation intensity plays a critical role and is commonly quantified using the cavitation number (C_v_)—a dimensionless parameter that reflects the propensity for cavitation to occur. The cavitation number is significantly influenced by the characteristics of the cavitation device, including its geometry, orifice diameter, and the pressure ratio across the device [15]. It is defined as:

$$Cavitation\;number\;\left(C_{V}\right)\;=\;\frac{\text{Static}\;\text{pressure}\;\text{head}}{\text{Dynamic}\;\text{pressure}\;\text{head}}$$

One dimensionless parameter is the cavitation number, and it is defined as:

$$C_{V}=\;\frac{P_{2}-P_{v}}{\frac{1}{2}\rho V^{2}}$$

Where,

P_2_ is the recovered pressure downstream of the cavitation device (Pa),
P_v_ is the liquid's vapor pressure at operating temperature (Pa),
V is the device's throat velocity (m/s), and
ρ is the density of water (kg/m^3^)

Cavitation-induced bubble creationoccurs at each hole's leading edge. The adjacent cavity is split due to the tendency of the inflow streams to approach the cavity surface and penetrate it. With a bit of vapor, the stream exits the MHO [11]. In a hydrodynamic cavitation reactor, the thickness of the orifice plate influences the formation and intensity of cavitation. Increasing the length of the flow constriction is necessary for a gradual pressure recovery downstream. Due to extended periods of low pressure, the cavitation inception pressure is often lower, resulting in a sharper transition and quicker pressure recovery. For quick pressure recovery, a thin orifice plate is appropriate. Higher downstream pressures may cause cavitation to begin, resulting in less cavitation [5]. The cavitation number indicates the extent of cavitation that occurs during the process. Cavitation usually happens when the C_V_ value falls below 1.0. In contrast, cavitation typically forms under a more significant value than 1.0 of C_V_ in many circumstances because of gas and suspended particles acting as cavitation nuclei. If the section is fixed, the inlet pressure determines the device's throat velocity, which is correlated with the cavitation number [15]. Lower cavitation numbers would result in a more significant quantum of cavitation. High cavitation intensity causes the bubbles to collapse, intensifying the dissociation of H_2_O molecules trapped inside the cavities and producing more •OH radicals.

### 2.2 Experimental setup

**Fig. 2:**
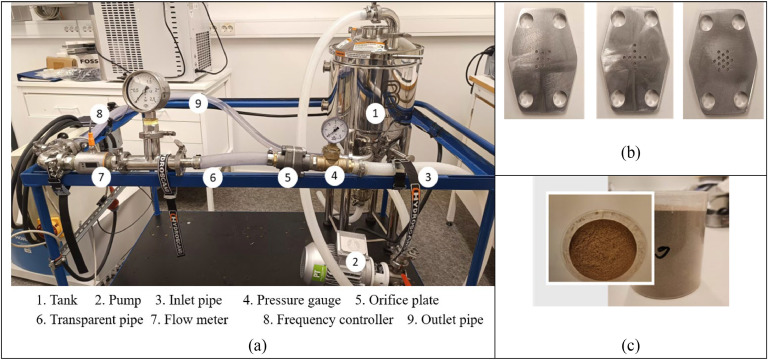
(a) Experimental setup of the Hydrodynamic cavitation process, (b) Orifice plates, and (c) Biomass particles.

This study experiment constructs an experimental setup. Fig. 2(a) displays the configuration of the experimental setup. A closed-loop system collects water from the tank, transfers it to the cavitation zone, and then returns the treated solution to the tank. The other main components of the system are a centrifugal pump, frequency control, control valves, a flow meter, and flanges that hold the orifice plate, tank, and pressure gauges. The flanges where the plates are to be mounted have a diameter of 25 mm. Water from the tank flows through a centrifugal pump during experimentation and is directed to the transparent section after the orifice plates, which creates hydrodynamic cavitation. A bypass line has been added to control the flow rate. While downstream pressure remains constant, the flow rate is changed to obtain various measurements. Continuous pressure was used to observe the effect.

In this study, hexagonal-shaped orifice plates with a thickness of 4 mm were made, which are adjustable within the flanges, however, the flow access area is a 25 mm diameter circular area. The bolt holes are 12 mm in diameter, and the effective cavitation holes are 2 mm in diameter. These dimensions make a single orifice plate. Three plates (case no 3, 5, and 7) are made for experimentation purposes; details of those plates are given in Table 4. The orifice plates used for the experimentation below show the four corner holes for the flange connection. To observe the effect of particles on cavitation, 200 g of wood (Oak) particles are mixed with 10 L of water as biomass. Wood was crushed and sieved using a crusher to obtain particles of 0.8 mm in size. After pouring the particles into the water, they are mixed perfectly by stirring. Then, the mixture circulates within the system. Figures 2(b) and 2(c) show the Orifice plates and particles used in the experiment, respectively. Only water also circulates to get the effect of particles in the process.

**Table 4:** Geometry of different orifice plates.

## 3 Results and analysis

A series of simulations on cases 1 to 14 (Table 4) analyzes the geometrical parameters, the spreading of the hole, MHO thickness, and the number of holes in the vapor phase generated from MHO. The effect of biomass particles on cavitation and particle density in the flow is studied through case studies and validated experimentally.

### 3.1 Computational fluid dynamics analysis

#### 3.1.1 Effect of orifice thickness

Plate thickness is one of the significant parameters affecting cavitation. The influence of orifice thickness in a dimensionless form, t/D, is investigated to generate cavitation. t and D define the plate thickness and plate diameter, respectively. Because of the same unit of this dimension, t/D is a dimensionless number. This parameter determines the maximum cavity size that can be reached before its collapse and is predictable. The residence length in the cavities' low-pressure area following nucleation is favorable for both growth and the pressure recovery zone. Multiple cavities may consolidate or disintegrate, depending on the level of contact with other cavities in the area [5]. The CFD model used t/*D* = 0.12, 0.16, 0.2, and 0.24. This observation is explained by the fact that each hole has fewer vena contracta effects because the thicker orifice gives the hole's contraction area more length, which lessens separation problems. Additionally, the orifice thickness impacts the turbulent kinetic energy for P_in_ = 300,000 Pa and P_r_ = 3 [11]. Among these geometries, t/*D* = 0.16 means 4 mm, showing the minimum cavitation number (Figure S2). For case no.5, according to the cavitation number, 4 mm is the optimized thickness among 3,4,5, and 6 mm of the orifice plate. These four cases have the same open area and hole orientation. As a flow nozzle, the issued jets have better guidance when the MHO thickness increases. However, due to the flow separation, the sharp edge at the entrance causes additional energy loss [27].

#### 3.1.2 Flow pressure

**Fig. 3:**
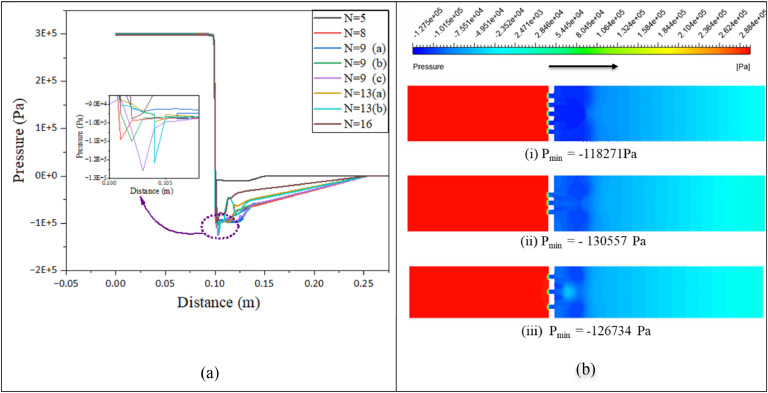
(a) Pressure vs Distance for all 4 mm thickness (b) Pressure contours (i) Case no.3 (ii) Case no 5 and (iii) Case no 7.

Cavitation intensity, the number of cavities, and collapse pressure depend on the sudden minimum pressure, inlet pressure, Pressure ratio (P_r_), system configuration, and pressure recovery length. A pressure difference creates turbulence immediately after the orifice plate. Fig. 3(a) presents the pressure distribution along the flow path for all 4 mm thickness plates mentioned in Table 4. The flow through the orifice increases as the inlet-to-exit pressure ratio rises, and the system's lowest pressure begins to fall.

The pressure differential between the HC reactor and the Cavitation number (C_v_) primarily determines the number of cavities that form and, therefore, the cavitation intensity [12]. The cavity forms due to a pressure fluctuation close to the throat. After analyzing these geometries, 3 mm and 6 mm thicknesses were also investigated for case 5. One factor that determines cavity dynamics is the rate of pressure recovery. Cavitation starts when the local low-pressure area created by separation gets close to the vapor pressure [11]. This study set the outlet absolute pressure at 100,000 Pa and examined the impact of input pressure for 300,000 Pa. In the graph of Fig. 3(a), the flow initially flows at 3000,000 Pa pressure without dropping the pressure. The inlet region shows the same curve for all geometries. At the orifice region, the pressure drops suddenly. The maximum pressure drop differs for different geometries, and the flow separates with the highest velocity in this region. Then, the pressure gets close to the vapor pressure, and cavitation starts. Figure 3(b) illustrates the pressure distribution of the flow. After the orifice plate, pressure recovery lengths are different for different geometries. The liquid velocity in the throat section produces higher shear stress at low cavitation numbers, resulting in a higher mass transfer rate at the optimum pressure. 300,000 and 400,000 Pa are considered the optimum inlet pressures for hydrodynamic cavitation applications [6]. Case no 5 shows the minimum pressure at the throat section within fourteen geometries. Case no 7 and case no 3 provide the nearest values to case no 5. For other geometries, the pressure drop is relatively low, indicating a lack of cavitation. Therefore, Figure 3(b) presents three geometry pressure contours, and others are added to Figure S4. In the graph, negative pressure is not absolute but static pressure. The pressure contours present the pressure intensity in the total flow path. When a sudden pressure drop at a water vapor pressure of 3540 Pa, water converts to vapor. Pressure increases after the cavitation reactor, and the bubble starts to collapse and release energy when the bubble exceeds the vapor pressure. This is a pressure recovery region; water bobbles are visible in that section. Zhi-Jiang Jin et al. investigated the cavitation phenomena under Pr = 3 for a single-hole orifice(t/*d* = 1); the plate found a minimum absolute pressure of 2645 Pa (static −98,680 Pa) [28]. This study found a minimum static pressure of −130,557 Pa for case no 5.

#### 3.1.3 Turbulence kinetic energy (TKE)

Information on the turbulence intensity behind the orifice plate can be helpful when assessing the dynamics of the bubbles produced by the multi-hole orifice. This turbulence intensity significantly impacts the subsequent mixing needed for the oxidation process's chemical reaction [11]. For all geometries (Table 4), Figure S5 presents the turbulence intensity. Here, the hole orientation turbulence intensity varies, even in cases where the open area, number of holes, and thickness are the same. Intensity increases with the number of holes, whereas cavitation and vapor fraction decrease for more than nine holes. Therefore, case no 5 shows the best result. Case no 3 and 7 show the nearest turbulence kinetic energy to case no.5. The turbulence intensity contours for different geometries are shown in Figure S6. When the impact of hole distribution on the output of turbulence intensity is evaluated, the asymmetric long-side circulation zone generates a high spot of turbulence intensity. Case no 5 generates 34.271 m^2^s^-2^, and case no 7 has 31.512 m^2^s^-2^, whereas both cases have the same open area and thickness. The effect of hole orientation on the plate has a significant impact on this matter. Higher turbulence accelerates the mixing of the fluids present after the orifice with biomass, which is a priority after cavitation phenomena for biomass pretreatment, such as delignification. Therefore, based on the sudden pressure drop and turbulence intensity observed in case no 5, the 4 mm thick plate with nine holes can be considered the optimized geometry.

#### 3.1.4 Throat velocity

**Fig. 4:**
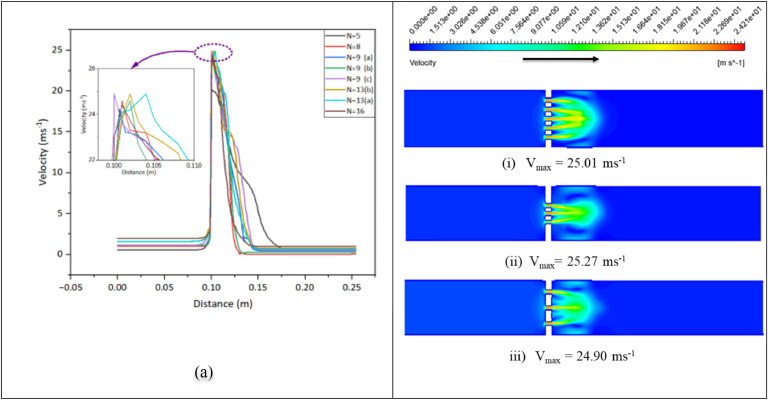
(a) Velocity vs Distance for thickness 4 mm (b) Velocity contours (i) Case no.3 (ii) Case no.5 (iii) Case no.7.

The throat velocity is inversely proportional to the pressure drop in the flow; the greater the pressure drop, the greater the velocity in the throat section. Higher velocity reduces the cavitation number, leading to increased cavitation, which is necessary for biomass pretreatment, such as delignification. Here, for all 4 mm thickness orifice geometries (Table 4), the velocity vs. distance relation of the flow is shown in Fig. 4(a), and others are shown in Figure S7.

For simplicity, the velocity distribution contour plots are shown in two dimensions. Several jets from the MHO are joined into a single jet, as seen in Figure 4(b). This released jet creates a lengthy, symmetrical circulation zone between the joined jet and the inner wall of the pipe. The jets' interaction improves in case number five compared with the others. As it approaches the center, a weak jet gives way to a stronger one. Cross-sectional area and velocity are inversely related for a constant total flow rate. According to Bernoulli’s principle, if the velocity of the fluid increases, the pressure tends to decrease [29]. Here, the maximum velocity obtained for case no 5 is 25.27 ms^-1^, whereas 25.01 ms^-1^ and 24.90 ms^-1^ for case no 3 and 7, respectively. Velocity and flow rate change for cases 3 and 5, even if both geometries have the same open area and thickness. This is due to the orientation of the orifice hole on the plates. In the study of Zhi-Jiang Jin et al. under pr = 3 for a single-hole orifice(t/*d* = 1) plate, they found a maximum velocity of 29 ms^-1^ [28]. This study found a maximum velocity of 25.27 ms-1 for case no 5 for pr=3. Figure 4(b) shows the velocity contours of cases no.3, 5, and 7, respectively, and others are added to Figure S8.

#### 3.1.5 Cavitation number

**Fig. 5:**
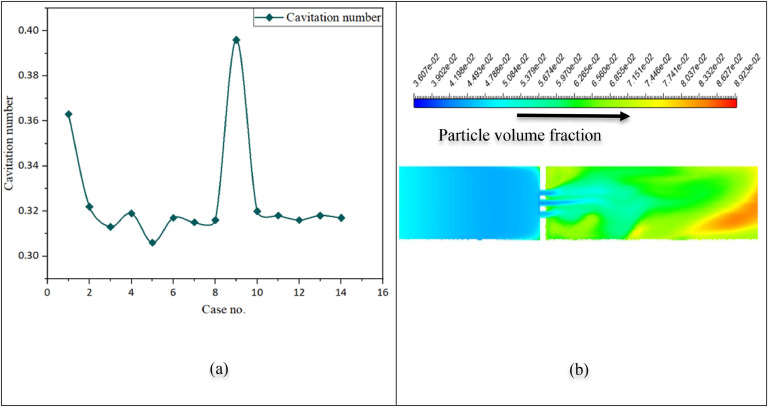
(a) Cavitation number for different geometries, (b) Volume fraction of particles in the flow.

In this study, the orifice diameter was maintained at 2 mm to ensure a smooth flow of slurry (a mixture of water and biomass) through the orifice plate. In Fig. 5(a), Case no 5 shows the minimum cavitation number compared with other geometries. The contraction ratio and orifice plate geometry directly correlate with the fluctuation amplitude. The cavitation number needs to be reduced to meet the requirement of higher cavitation. Cases no 5,6, and 7 have the same thickness and hole number. Even if these plates have the same open area, the cavitation number changes, as shown in Figure 5(a). This change happens due to the hole orientations on the plate. According to the minimum cavitation number, case no.5 is the optimized geometry.

#### 3.1.6 Cavitation with biomass particle

Particles break in two ways: fracture and abrasion. Cavitation, which is dominated by the development of temporary cavitation bubbles, results in more abrasion and the generation of smaller particles [30]. The three-phase numerical flow analysis uses liquid water, water vapor, and biomass particles as the first (primary), second, and third phases. Biomass particles are the flow's third (secondary) phase with 5 % V/V [31]. The biomass density is less than that of water, even less than half. So, during biomass treatment, biomass particles may create a blockage in the orifice region. Variations in the cavitation number could be observed. Different cavitation numbers indicate different amounts of cavitation bubbles. For cases 3, 5, and 7, cavitation phenomena were investigated using three phases, and the difference between cavitation in water only and particle-mixed water cavitation in the same cavitation generation unit was analyzed. The biomass volume fraction contour shows the particle density in the flow in Figure 5(b). Here,5 % (v/v) or 2 % (W/W) of wood particles are used as biomass particles. The concept of an interpenetrating continuum serves as the basis for the Euler-Euler formulation. A different set of mass and momentum conservation equations is solved for every phase. With the dispersion flow, the impact of the particles on cavitation formation is observed. The volume proportion of particles in the mixture determines the solid shear viscosity [32]. Due to gravity, wood particles move upward after the orifice. Wood particles' physical density is lower than that of water. Therefore, wood particles try to move up in the flow. In the throat section, the presence of particles in the flow is low due to the high fluid velocity. Wood particles with a density of400 kg/m^3^ are used here as a granular third phase. The presence of particles affects the turbulence intensity. The max throat velocity decreases from 25.27 ms^-1^ for only water to 23.42 ms^-1^ for particle mixture flow in case no.5, so the cavitation number increases. The cavitation number is 0.36 for the particle mixture flow, whereas it is 0.31 for the water flow only at an inlet pressure of 300,000 Pa. An increase in the cavitation number means less cavitation and bubble generation, which is the effect of particles on the cavitation process.

### 3.2 Experimental analysis

This study employed a transparent acrylic pipe to visualize cavitation phenomena. Cavitation bubble formation was observed immediately downstream of the orifice plate. The bubbles formed as the local pressure dropped below the vapor pressure and collapsed when exposed to higher downstream pressures. A variable frequency drive was used to control the flow rate by adjusting the pump operating frequency. The pump initially operated at 20 Hz, and the frequency was gradually increased to 50 Hz in increments, corresponding to the pump's maximum power output.

At lower frequencies (and thus lower inlet pressures and flow rates), cavitation was first detected, allowing the identification of key influencing parameters. Inlet pressure and flow rate exhibited initial fluctuations, stabilizing after 2–3 min of continuous operation. Thereafter, both parameters increased gradually. Cavitation was monitored using a pressure ratio defined by the inlet pressure (P_in_) and outlet pressure (P_out_), measured by pressure gauges installed on either side of the orifice plate. A flowmeter was used to calculate the total flow rate through the pipe. Since fluid temperature significantly affects cavitation characteristics, a handheld infrared thermometer was employed to monitor and maintain the working fluid at approximately 30 °C throughout the experiments which utilized hot water.

#### 3.2.1 Cavitation for only water

**Fig. 6:**
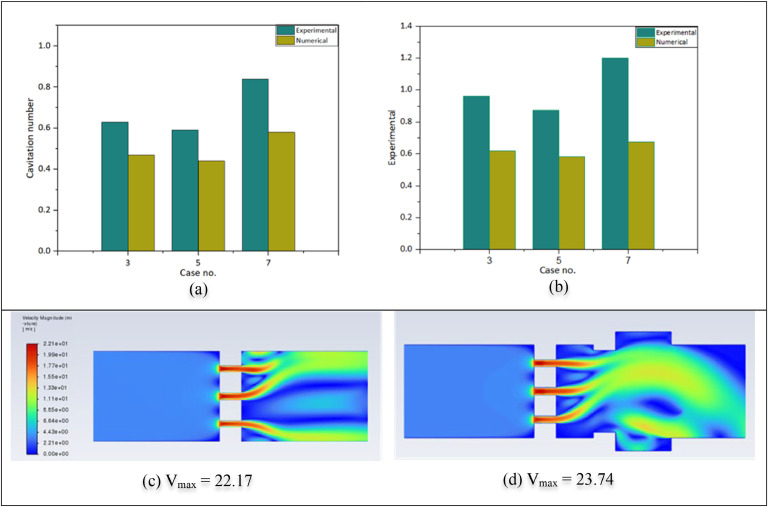
Experimental and Numerical cavitation number (a) for water, (b) for the mixture; and Velocity contour (c) for the straight pipeline, (d) coupling pipeline.

Orifice plates with various dimensions are used in the same experiment, and cavitation phenomena were investigated by changing one by one for cases 3, 5, and 7. The cavitation bubbles and flow are observed. A smaller cross-sectional flow area produces a higher orifice velocity and, thus, a lower Cavitation Number when the downstream pressure remains constant. A bypass line redirected the pump's flow to change the flow rate. It is important to note that the results show a difference in the hole orientations on the plate for the same flow area and number of holes. That is also numerically shown and analyzed. In the experiment, the inlet pressure differs for three geometries. For case no 3 and 5, the inlet pressure are 190,000 and 180,000 Pa, respectively, whereas it is 140,000 Pa for case no 7. The numerical analysis investigates all simulations with an inlet pressure of300000 Pa. For validation, these three geometry cavitations are investigated numerically using similar experimental inlet pressures, such as 190,000, 180,000, and 140,000 Pa. Fig. 6(a) presents the cavitation number for only water flow.

#### 3.2.2 Cavitation for particle mixture

All orifice plates had a fixed 2 mm diameter. This study focused on cavitation for biomass pretreatment, so the orifice size did not change to biomass particle size. Considering sedimentation and floating particles, the particles are mixed well by stirring. Particles may tend to block in the cavitation reactor section; therefore, 0.8 mm particles are used with water. Particles of any size <2 mm can be used here. For cases no.5 and 7, experimentation is investigated. Figure 6(b) presents the values and their differences. Initially, the flow fluctuated, but after running the system for a few minutes, it became almost constant at a 50 Hz frequency over time. Then, the outlet pressure is kept at atmospheric pressure, and the 30 °C mixture temperature, flow rate, and pressures were measured. Here, numerical and experimental values differ, but their trend is the same. Cases 3 and 5 have the same open area, but the cavitation is different for the mixture as well.

The Experimental and numerical values differ by around 30 % for only water and >30 % for the particle mixture. Two reasons for this difference are described here.

○ **Pressure loss**: In the experimental setup, pressure losses were present due to the elbow, height, coupling difference, and inner diameter difference between the pipe and joint. These losses were not considered in the numerical analysis. Moreover, in the numerical analysis, only the cavitation reactor part, which means the orifice plate and pipe, was considered instead of the whole setup. Fig. 6(c) shows the velocity for a straight pipeline,& figure 6(d) shows the velocity profile for a pipeline with coupling. Changing velocity means cavitation changes. The first one has a straight pipe similar in diameter to the orifice plate. At the same time, the other one has a coupling after the orifice plate. Changes in inner diameter fluctuate the pressure and velocity. Therefore, the occurrence of cavitation became different.
○ **Maximum velocity calculation:** Depending on the flow velocity, density, and viscosity, a pipe's flow can be laminar or turbulent. The local velocities in cylindrical conduits are precisely parallel to the conduit's axis, the layers are cylindrical, and their velocities increase parabolically from zero at the wall to a maximum at the centre. The destruction and mixing of the layers in a laminar flow, along with chaotic or turbulent local motions in the fluid, are characteristics of this flow. This type of flow is referred to as turbulent flow. While local velocity at the pipe wall remains zero, the axial velocity distribution in circulator conduits is more uniform than in laminar flow [29]. The maximum velocity is needed to calculate the cavitation number of a cavitation reactor. It depends on the maximum velocity. The maximum velocity can be obtained in numerical analysis, but it needs to be calculated using the flow rate and cross-sectional area. Figure S9 shows the flow velocity profile of the orifice hole cross-section.

Flowrate,Q=VA

Q=Flowrate

A=Openarea

This study uses the maximum throat velocity when calculating the cavitation number for numerical analysis. An experiment calculates this velocity from the flow rate and open area. Therefore, the velocity is calculated from the flow rate, resulting in an average velocity that differs between the peripheral and central holes. The numerically used velocity is the maximum from the central hole. For this reason, a discrepancy exists between the numerical and experimental results. Experimental velocity is less than numerical velocity. For this velocity difference, the cavitation number has changed.

For future studies, straight joints can effectively mitigate the pressure drop within the system. Unlike adapter joints, straight connections do not introduce diameter variations that contribute to additional energy losses. In contrast, adapter joints inherently cause diameter changes, increasing pressure drop, particularly within the joining region. A flow sensor allows accurate velocity detection for velocity measurements in the orifice hole region. The reduced pressure drop and precise velocity measurement can minimize the observed discrepancy of approximately 30 % in cavitation numbers between experimental and numerical results.

## 4 Conclusion

This study utilized MHO as a cavitation reactor to generate bubbles. However, most computational fluid dynamics (CFD) models in previous studies have focused solely on water, neglecting the influence of particles within the mixture during biomass pretreatment or water treatment. This work investigated the impact of orifice plate geometrical parameters—namely, thickness (3–6 mm), number of holes (5–16), and hole orientation—on cavitation performance for both water and biomass-water mixtures. Numerical simulations were conducted to determine the optimal configuration based on pressure drop, throat velocity, and cavitation number. Among the tested geometries, a 4 mm plate thickness, nine holes, and the hole orientation of case 5 yielded the most favourable cavitation conditions, characterized by a minimum pressure drop, a low cavitation number (Cv = 0.44), maximum turbulence, and a high vapour volume fraction. Subsequently, three selected cases were simulated using a biomass mixture containing wood particles as the working fluid. The presence of biomass altered flow behaviour significantly: in case 5, turbulence intensity decreased from 34.27 m²/s² (water alone) to 19.512 m²/s² (biomass mixture), and the cavitation number increased from 0.46 to 0.59, indicating reduced cavitation under an inlet pressure of 300,000 Pa. Experimentation was performed for cases 3, 5, and 7 by measuring water and biomass mixtures' flow rate and inlet and outlet pressure. Consistent with the simulation, case 5 exhibited the lowest cavitation number (Cv = 0.59) among the tested configurations, confirming its optimal performance. The experimentally measured cavitation number for the biomass mixture in case 5 was 0.93, further substantiating the reduced cavitation effect in the presence of biomass despite some quantitative differences between simulation and experimental results. The presence of biomass has been found to mitigate the cavitation phenomenon. The particle size of the biomass plays a key role in defining the characteristics of the mixture. The present study used biomass particles with an average size of 0.8 mm to prepare the slurry. Using a finer biomass powder in the slurry formulation could further enhance the cavitation performance of the reactor. However, scaling up cavitation-based pretreatment requires careful regulation of cavitation intensity to achieve uniform treatment of biomass slurries under high-throughput conditions. Slurry properties, including viscosity, solid loading, and particle size distribution, strongly influence cavitation behavior, and ensuring consistent performance at larger scales remains a significant engineering challenge. Nevertheless, recent advancements in reactor design and process intensification indicate that continuous cavitation-assisted pretreatment holds promise for industrial application, provided that energy efficiency and operational stability are adequately optimized.

### 4.1 Recommendations

As the flow is a water mixture, corrosion is a factor in the smooth running of the process. Mild steel is corrosive, while stainless steel and aluminum are non-corrosive. In the case of cavitation bobble penetration, stainless steel is more rigid than aluminum. Future investigations could involve the interaction between cavitation bubbles and the orifice plate and factors like plate deformation and vibrations to consider the erosion or fatigue of the orifice plate/pipe with varying materials including steel and aluminum.

## CRediT authorship contribution statement

**Shetal Roy:** Writing – review & editing, Writing – original draft, Visualization, Validation, Software, Methodology, Investigation, Formal analysis, Data curation. **Souman Rudra:** Writing – review & editing, Validation, Supervision, Project administration, Methodology, Investigation, Formal analysis, Conceptualization. **DI Muhandiram:** Writing – review & editing, Supervision, Methodology, Investigation. **MM Roshid:** Writing – review & editing.

## Declaration of competing interest

The authors declare the following financial interests/personal relationships which may be considered as potential competing interests:Souman Rudra reports financial support was provided by Norwegian Directorate for Higher Education and Skills (HK-Dir). Reports a relationship with that includes:. Has patent pending to. If there are other authors, they declare that they have no known competing financial interests or personal relationships that could have appeared to influence the work reported in this paper.

## Data Availability

Data will be made available on request.

## Appendix Supplementary materials

Image, application 1
