# Supplementary material for: Numerical modelling and experimental validation of an orifice plate-based hydrodynamic cavitation process for improving the biomass pretreatment
Source: Biotechnol Rep (Amst). 2025 Sep 12;48:e00925. doi: 10.1016/j.btre.2025.e00925 (PMC12482639; doi:10.1016/j.btre.2025.e00925)
Supplement: Supplementary file 1 [file mmc1.docx]

**Appendices**


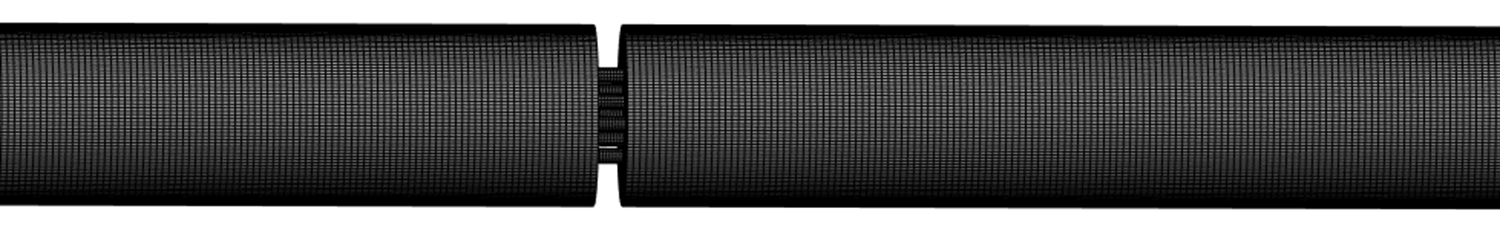


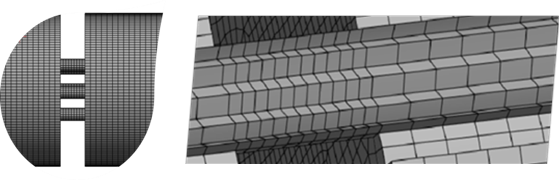


Figure S1: Mesh grid of the fluid domain.


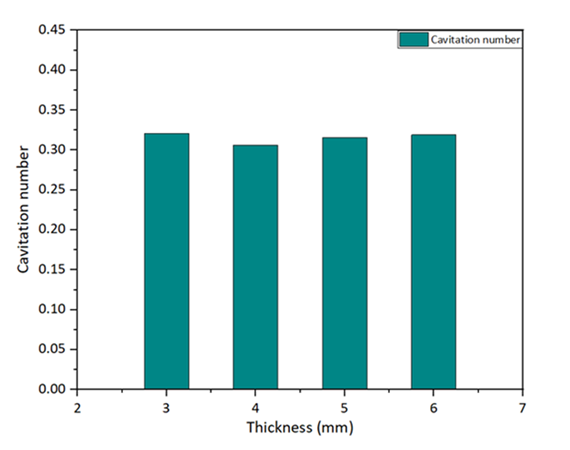


Figure S2: Cavitation number vs Plate thickness.


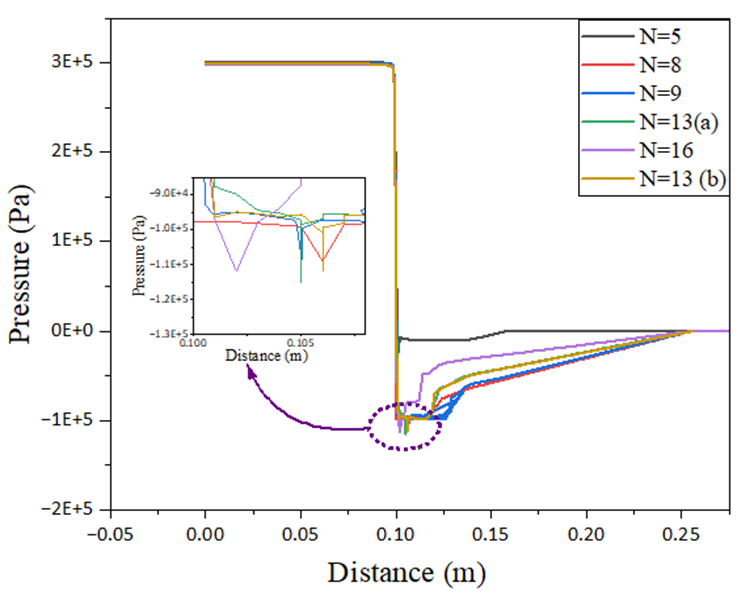


Figure S3: Pressure vs Distance for thickness 5mm plate.


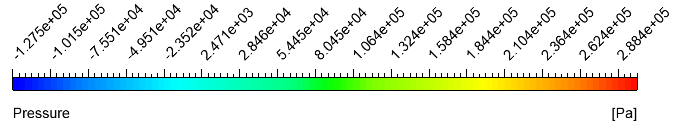


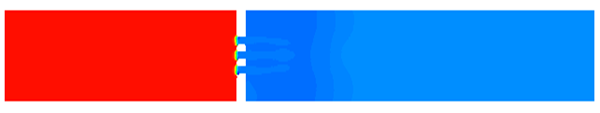


(C.1) P_min_= -44902.7Pa


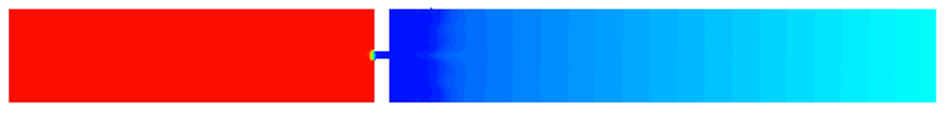


(C.2) P_min_= -109581Pa


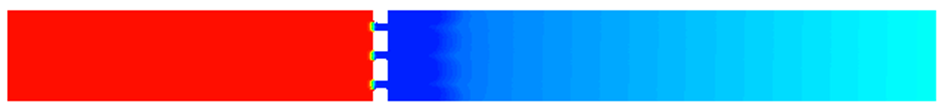


(C.4) P_min_= -110808 Pa


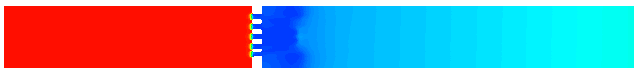


(C.6) P_min_=-11713Pa


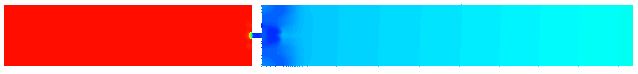


(C.8) P_min_=-114702Pa


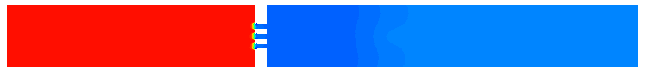


(C.9) P_min_=-39398Pa


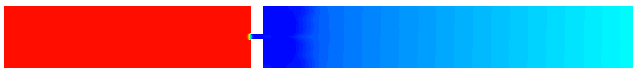


(C.10) P_min_=-105188Pa


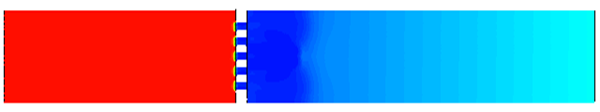


(C.11) P_min_= 109312Pa


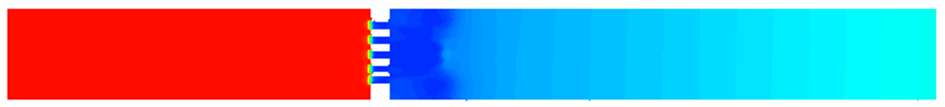


(C.12) P_min_= -115636Pa


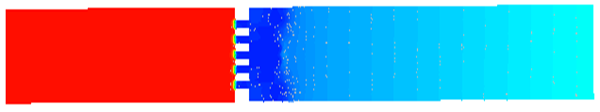


(C.13) P_min_=-111535Pa


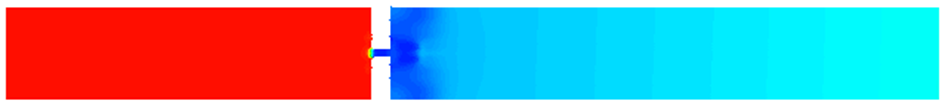


(C.14) P_min_= -112901Pa

Figure S4: Pressure contours of different case.


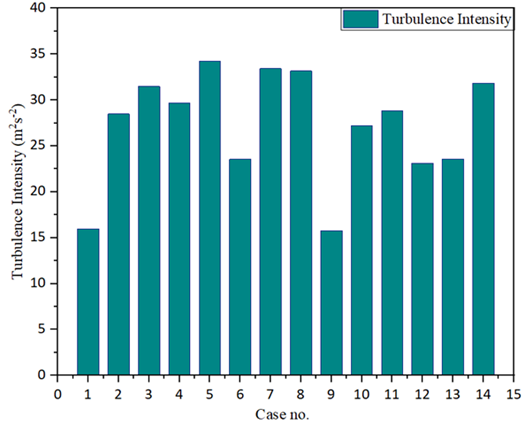


Figure S5: Turbulence intensity for all geometries.


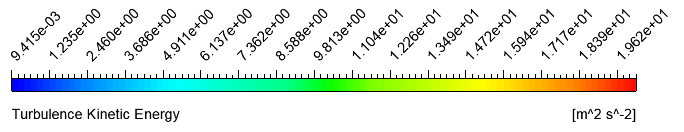


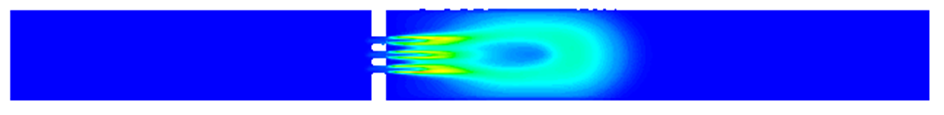


(C.1) TKE_max_= 15.97m^2^s^-2^


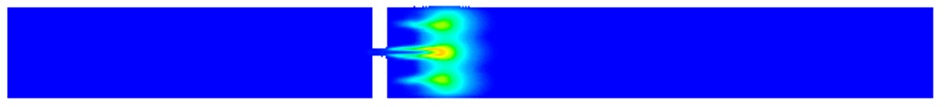


(C.2) TKE _max_= 28.5 m^2^s^-2^

^
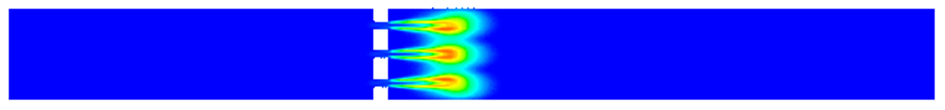
^

(C.4) TKE_max_= 29.7 m^2^s^-2^

^
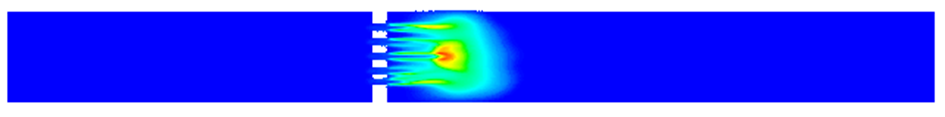
^

(C.6) TKE_max_= 23.55m^2^s^-2^

^
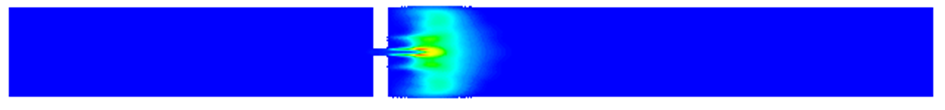
^

(C.8) TKE_max_=33.42 m^2^s^-2^

^
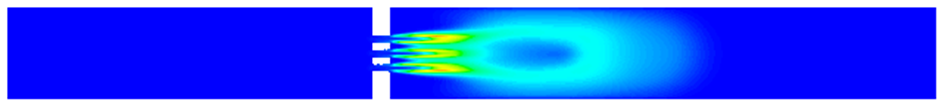
^

(C.9) TKE_max_= 15.79m^2^s^-2^

^
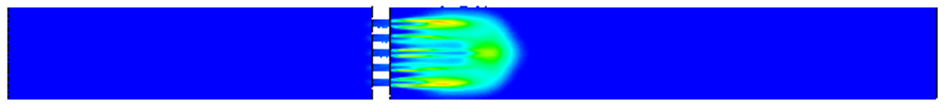
^

(C.10) TKE_max_= 21.19m^2^s^-2^


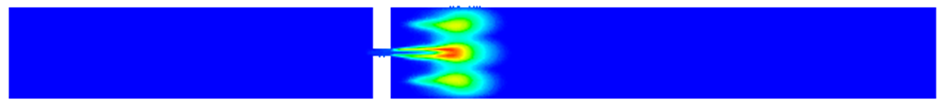


(C.11) TKE _max_= 20.23 m^2^s^-2^


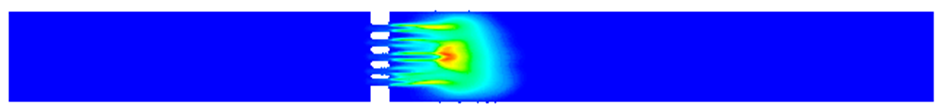


(C.12) TKE_max_= 23.11m^2^s^-2^

^
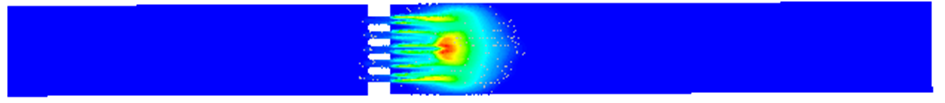
^

(C.13) TKE _max_= 23.43 m^2^s^-2^

^
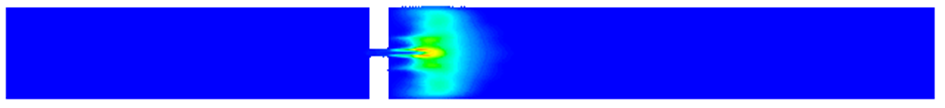
^

(C.14) TKE _max_=31.94 m^2^s^-2^

Figure S6: Turbulence Kinetic Energy contours of different case.


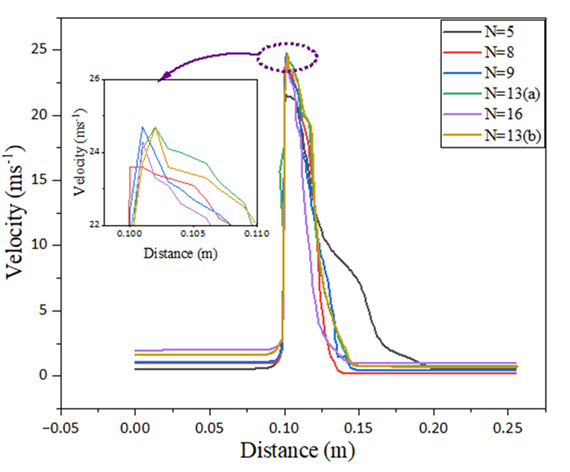


Figure S7: Velocity vs Distance for thickness 5mm.


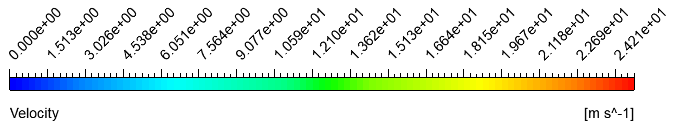


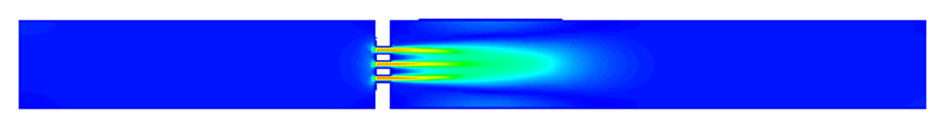


(C.1) V_max_= 24.76ms^-1^

^
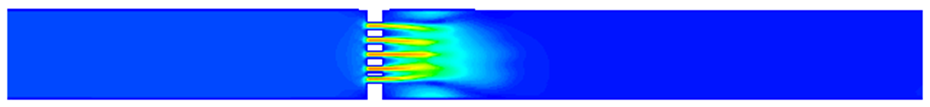
^

(C.2) V_max_= 24.81 ms^-1^


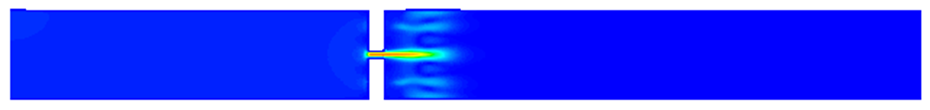


(C.4) V_max_= 24.78 ms^-1^

^
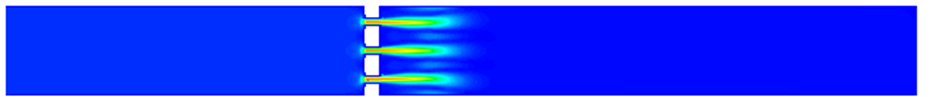
^

(C.6) V_max_= 24.93ms^-1^

^
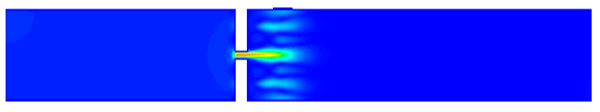
^

(C.8) V_max_= 24.9 ms^-1^

^
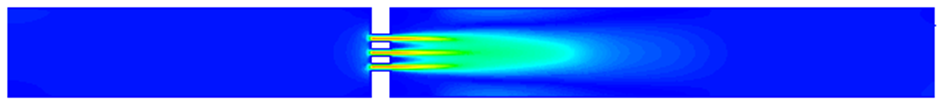
^

(C.9) V_max_= 22.21 ms^-1^


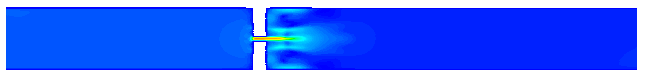


(C.10) V_max_= 24.72 ms^-1^


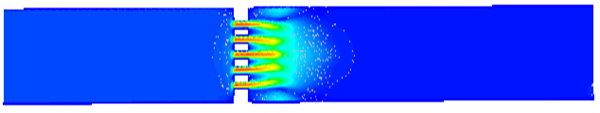


(C.11) V_max_= 24.84 ms^-1^


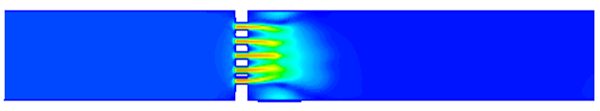


(C.12)V_max_= 24.86 ms^-1^


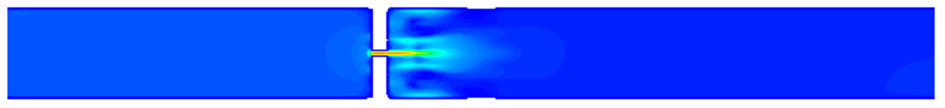


(C.13) V_max_ = 24.27 ms^-1^

Figure S8: Velocity contours of different case.

| 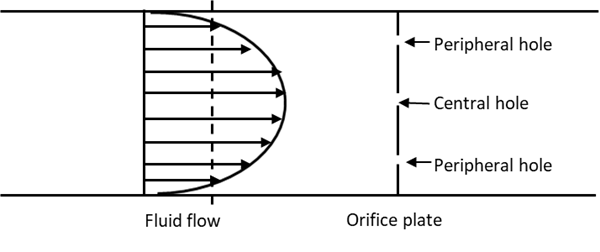 | Flow rate, Q = VA  Q= Flow rate  A=Open area |
| --- | --- |

Figure S9: Fluid flow through an MHO.
